# Antioxidative, Antiapoptotic, and Anti-Inflammatory Effects of Apamin in a Murine Model of Lipopolysaccharide-Induced Acute Kidney Injury

**DOI:** 10.3390/molecules25235717

**Published:** 2020-12-03

**Authors:** Jung-Yeon Kim, Jaechan Leem, Kwan-Kyu Park

**Affiliations:** 1 Department of Immunology, School of Medicine, Catholic University of Daegu, Daegu 42472, Korea; jy1118@cu.ac.kr; 2 Department of Pathology, School of Medicine, Catholic University of Daegu, Daegu 42472, Korea

**Keywords:** acute kidney injury, sepsis, apamin, lipopolysaccharide, oxidative stress, apoptosis, inflammation

## Abstract

Sepsis is the major cause of acute kidney injury (AKI) in severely ill patients, but only limited therapeutic options are available. During sepsis, lipopolysaccharide (LPS), an endotoxin derived from bacteria, activates signaling cascades involved in inflammatory responses and tissue injury. Apamin is a component of bee venom and has been shown to exert antioxidative, antiapoptotic, and anti-inflammatory activities. However, the effect of apamin on LPS-induced AKI has not been elucidated. Here, we show that apamin treatment significantly ameliorated renal dysfunction and histological injury, especially tubular injury, in LPS-injected mice. Apamin also suppressed LPS-induced oxidative stress through modulating the expression of nicotinamide adenine dinucleotide phosphate oxidase 4 and heme oxygenase-1. Moreover, tubular cell apoptosis with caspase-3 activation in LPS-injected mice was significantly attenuated by apamin. Apamin also inhibited cytokine production and immune cell accumulation, suppressed toll-like receptor 4 pathway, and downregulated vascular adhesion molecules. Taken together, these results suggest that apamin ameliorates LPS-induced renal injury through inhibiting oxidative stress, apoptosis of tubular epithelial cells, and inflammation. Apamin might be a potential therapeutic option for septic AKI.

## 1. Introduction

Acute kidney injury (AKI) is characterized by a sudden decline in renal function [1]. Although there are numerous potential causes of AKI, sepsis is a primary cause of AKI in patients admitted to intensive care units. Septic AKI worsens prognosis and increases mortality in severely ill patients [1,2]. However, although several nonspecific and reactive therapies have been used, there are still no therapies specifically targeting renal dysfunction and tubular injury in septic AKI in clinical settings. Therefore, it is necessary to discover and develop novel therapeutic agents for septic AKI. The pathogenesis of septic AKI remains incompletely understood, but several pathological processes, including oxidative stress, apoptosis of tubular epithelial cells, and inflammation, have been recognized as key players in the mechanisms of septic AKI [2,3]. During sepsis, pathogen-associated molecular patterns (PAMPs) are released into the bloodstream. Among them, lipopolysaccharide (LPS), an endotoxin produced by gram-negative bacteria, strongly activates renal tubular epithelial cells and pro-inflammatory cells via interaction with toll-like receptor 4 (TLR4) [2,3]. Activated cells secrete marked amounts of cytokines and thereby induce oxidative stress, tubular cell apoptosis, and inflammatory responses.

Recently, active substances obtained from natural products have been in the spotlight as new drug candidates [4,5]. Bee products have long been widely used due to their nutritional and health benefits [6,7]. Among them, bee venom is one of the most effective agents in traditional medicine, especially in Asia, to treat various human diseases [8]. Bee venom contains various active enzymes and peptides such as melittin and apamin. Typically, bee venom is collected from cultured honey bees using a collector device in a sterile manner, and various components are then extracted from it [9]. Much research has focused on the biological effects and clinical potential of bee venom and its main component, melittin [10,11,12]. However, accumulating evidence also has highlighted the importance of apamin as a drug candidate [13]. Apamin has been shown to exert anti-inflammatory, antioxidative, and antiapoptotic activities in animal models of several inflammatory diseases, including gouty arthritis [14], multiple sclerosis [15], chronic liver disease [16], acute pancreatitis [17], and atherosclerosis [18]. However, no study has been conducted to evaluate whether apamin has a protective effect on LPS-induced AKI. Therefore, we investigated the potential effects of apamin on LPS-induced renal injury and explored the mechanisms involved in oxidative stress, apoptosis, and inflammation.

## 2. Results

### 2.1. Apamin Ameliorated LPS-Induced Renal Dysfunction and Histological Injury

**Figure 1:**
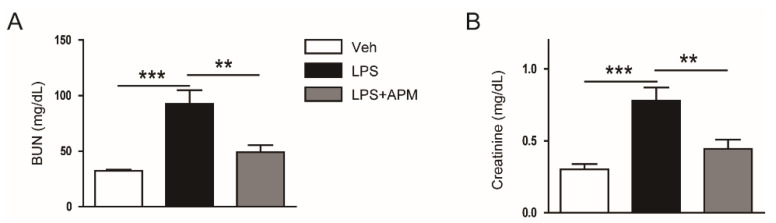
Effect of apamin on renal function in lipopolysaccharide (LPS)-treated mice. Apamin (APM; 0.1 mg/kg) was administered intraperitoneally to mice 1 h after LPS injection (10 mg/kg). (**A**) Blood urea nitrogen (BUN) levels. (**B**) Plasma creatinine levels. *n* = 8 per each group. ** *p* < 0.01 and *** *p* < 0.001.

Plasma creatinine and blood urea nitrogen (BUN) levels were increased after LPS injection (Figure 1A,B). However, post-treatment with apamin significantly reduced the increase in both levels (Figure 1A,B), suggesting that apamin alleviated the LPS-induced acute renal failure.

**Figure 2:**
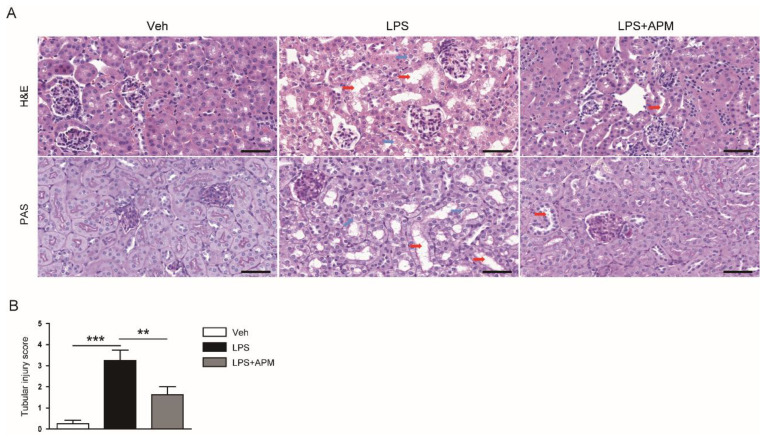
Histological examination of kidneys from all experimental groups. (**A**) Hematoxylin and eosin (H&E) and periodic acid Schiff (PAS) staining of kidney tissues. Red arrows indicate dilated tubules. Blue arrows indicate swelling of tubular epithelial cells. Scale bars: 50 μm. (**B**) Tubular injury score. *n* = 8 per each group. ** *p* < 0.01 and *** *p* < 0.001.

Next, hematoxylin and eosin (H&E) and periodic acid Schiff (PAS) staining was carried out on kidney sections from all experimental groups to investigate the effect of apamin on structural damage caused by LPS. We observed histological abnormalities, such as dilatation of tubules and swelling of epithelial cells, in LPS-injected mice (Figure 2A,B). However, this detrimental effect of LPS was significantly attenuated by apamin (Figure 2A,B).

**Figure 3:**
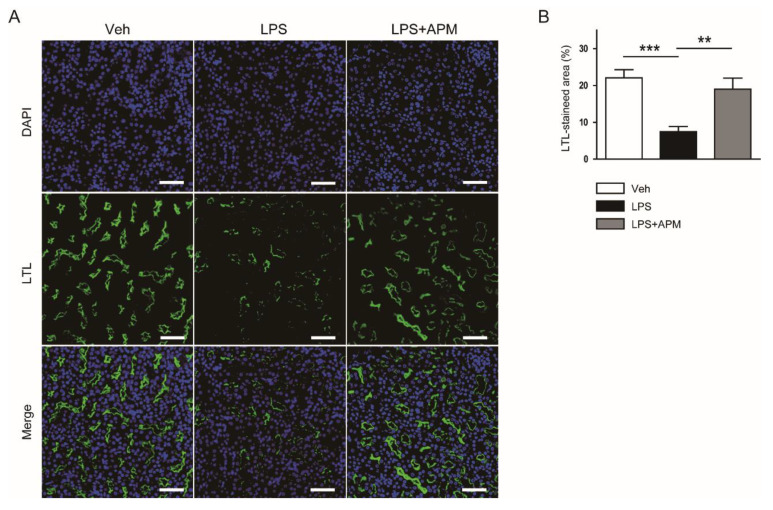
Effect of apamin on LPS-induced loss of brush borders in the proximal tubule. (**A**) Immunofluorescent staining with fluorescein isothiocyanate-conjugated lotus tetragonolobus lectin (LTL) of kidney tissues. Scale bars: 50 μm. (**B**) Percentage of stained areas for LTL. *n* = 8 per each group. ** *p* < 0.01 and *** *p* < 0.001.

Brush border loss in the proximal tubule is one of the hallmarks of AKI [2,3]. Thus, we stained the kidney sections with fluorescein isothiocyanate (FITC)-conjugated lotus tetragonolobus lectin (LTL) to observe the brush border of proximal tubule. LTL is a well-known marker for the brush border of proximal tubule [18]. We found that LPS injection largely increased the LTL-stained area, which was significantly reduced by apamin (Figure 3A,B). This finding suggests that apamin alleviates LPS-induced loss of brush borders in the proximal tubule.

**Figure 4:**
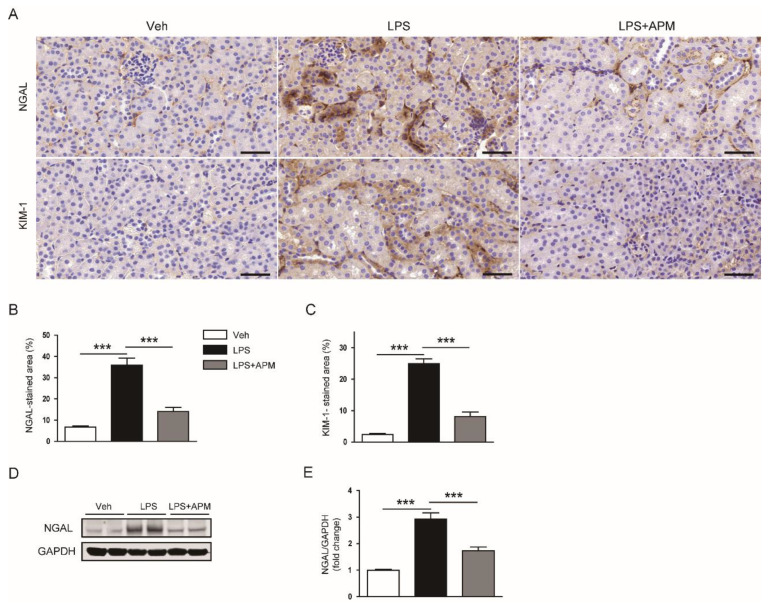
Effect of apamin on expression of tubular injury markers in LPS-injected mice. (**A**) Immunohistochemical staining of kidney tissues for neutrophil gelatinase-associated lipocalin (NGAL) or kidney injury molecule-1 (KIM-1). Scale bars: 50 μm. (**B**) Percentage of stained areas for NGAL. (**C**) Percentage of stained areas for KIM-1. (**D**) Immunoblotting of NGAL. (**E**) Quantification of immunoblot for NGAL. *n* = 8 per each group. *** *p* < 0.001.

To further evaluate the effect of apamin on tubular injury, immunohistochemial staining of kidney sections was performed with anti-neutrophil gelatinase-associated lipocalin (NGAL) or anti-kidney injury molecule-1 (KIM-1) antibody. We found that elevated expression of these tubular injury markers in LPS-injected mice was attenuated by apamin (Figure 4A–C). The suppressive effect of apamin on NGAL expression was also confirmed by immunoblotting (Figure 4D,E). Altogether, these results suggest that the administration of apamin after LPS injection ameliorates renal dysfunction and histological injury.

### 2.2. Apamin Suppressed LPS-Induced Oxidative Stress

**Figure 5:**
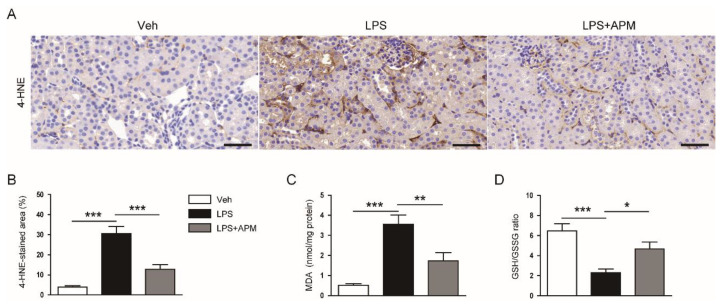
Effect of apamin on LPS-induced renal oxidative stress. (**A**) Immunohistochemical staining of kidney tissues for 4-hydroxynonenal (4-HNE). Scale bars: 50 μm. (**B**) Percentage of stained areas for 4-HNE. (**C**) Renal malondialdehyde (MDA) levels. (**D**) reduced glutathione (GSH)/oxidized glutathione (GSSG) ratio. *n* = 8 per each group. * *p* < 0.05, ** *p* < 0.01, and *** *p* < 0.001.

Oxidative stress plays an important role in septic AKI [2,3]. Previous studies have shown that there is a marked increase in oxidative damage in LPS-induced renal injury [19,20]. Thus, we examined the effect of apamin on LPS-induced oxidative stress. Immunohistochemical staining of kidney sections with an antibody against the lipid peroxidation product 4-hydroxynonenal (4-HNE) showed that the number of cells stained with 4-HNE in LPS-injected mice was significantly reduced by apamin (Figure 5A,B). In addition, renal levels of MDA, another reliable marker to assess lipid peroxidation, were reduced by apamin (Figure 5C). We also observed that the reduced glutathione (GSH)/oxidized glutathione (GSSG) ratio, an indicator of oxidative stress, was decreased in the kidneys of LPS-injected mice, which was significantly reversed by apamin (Figure 5D).

**Figure 6:**
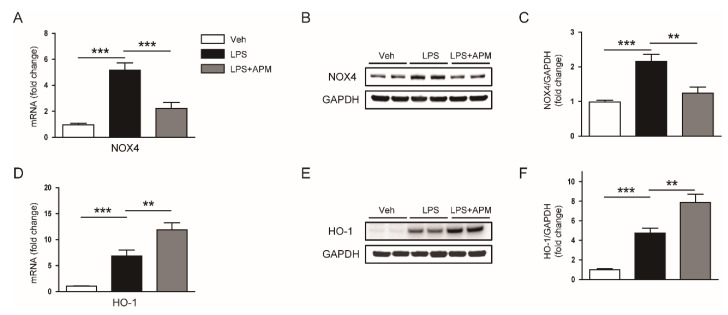
Effect of apamin on nicotinamide adenine dinucleotide phosphate oxidase 4 (NOX4) and heme oxygenase-1 (HO-1) expression. (**A**) The mRNA expression of NOX4. (**B**) Immunoblotting of NOX4. (**C**) Quantification of immunoblot for NOX4. (**D**) The mRNA expression of HO-1. (**E**) Immunoblotting of HO-1. (**F**) Quantification of immunoblot for HO-1. *n* = 8 per each group. ** *p* < 0.01 and *** *p* < 0.001.

Nicotinamide adenine dinucleotide phosphate oxidase 4 (NOX4) produces reactive oxygen species (ROS) and contributes to LPS-induced oxidative stress in the kidney [21,22]. In addition to the pro-oxidant enzyme NOX4, heme oxygenase-1 (HO-1) also plays an important role as a stress-inducible antioxidant enzyme in oxidative stress caused by LPS [23,24]. We found that an increase in renal mRNA and protein levels of NOX4 after LPS injection was significantly reduced by apamin (Figure 6A–C). We also observed that mRNA and protein levels of HO-1 were largely increased after LPS injection, which was further enhanced by apamin (Figure 6D–F). Altogether, these results suggest that apamin alleviates LPS-induced oxidative stress, at least partially, by modulating expression of NOX4 and HO-1.

### 2.3. Apamin Inhibted LPS-Induced Apoptosis

**Figure 7:**
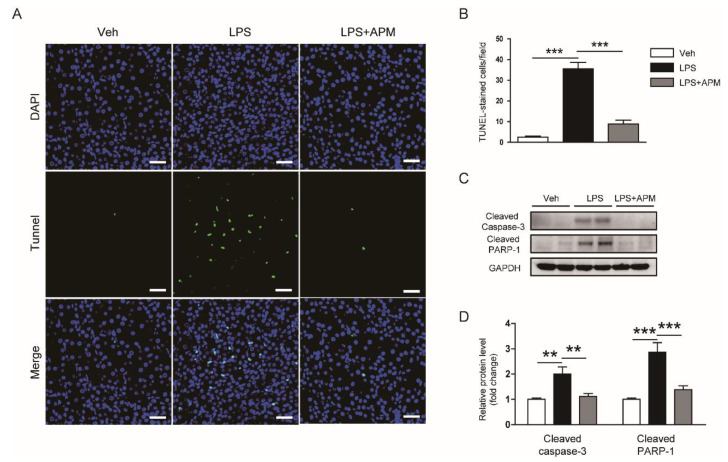
Effect of apamin on LPS-induced apoptosis. (**A**) Terminal deoxynucleotidyl transferase-mediated deoxyuridine triphosphate nick end labeling (TUNEL) staining of kidney tissues. Scale bars: 25 μm. (**B**) Number of cells stained with TUNEL. (**C**) Immunoblotting of cleaved caspase-3 and cleaved poly(ADP-ribose) polymerase-1 (PARP-1). (**D**) Quantification of immunoblots for cleaved caspase-3 and cleaved PARP-1. *n* = 8 per each group. ** *p* < 0.01 and *** *p* < 0.001.

Oxidative stress can induce renal tubular cell apoptosis [2,3]. Thus, we next performed TdT-mediated dUTP nick end labeling (TUNEL) assay to examine the effect of apamin on apoptotic cell death in LPS-induced AKI. LPS treatment largely increased the number of cells stained with TUNEL in the kidneys (Figure 7A,B). However, this change was significantly attenuated by apamin (Figure 7A,B). In addition, apamin reduced the elevated protein levels of cleaved forms of caspase-3 and poly (ADP-ribose) polymerase-1 (PARP-1) in LPS-injected mice (Figure 7C,D). These results suggest that apamin attenuates LPS-induced apoptotic death of tubular epithelial cells.

### 2.4. Apamin Suppressed LPS-Induced Inflammation

**Figure 8:**
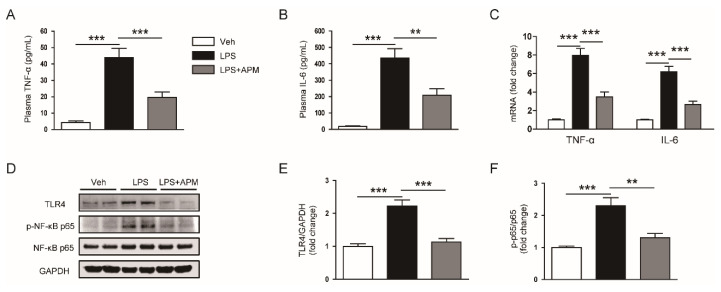
Effect of apamin on plasma and renal levels of cytokines, toll-like receptor 4 (TLR4) expression, and nuclear factor-κB (NF-κB) p65 phosphorylation in LPS-injected mice. (**A**) Plasma tumor necrosis factor-α (TNF-α) levels. (**B**) Plasma interleukin-6 (IL-6) levels. (**C**) The mRNA expression of TNF-α and IL-6 in kidney tissues. (**D**) Immunoblotting of TLR4, p-NF-κB p65, and NF-κB p65. (**E**) Quantification of immunoblot for TLR4. (**F**) Quantification of immunoblot for p-NF-κB p65. *n* = 8 per group. ** *p* < 0.01 and *** *p* < 0.001.

LPS activates renal tubular epithelial cells and immune cells via interaction with TLR4 [2,3]. We found that administration of apamin reduced circulating levels of tumor necrosis factor-α (TNF-α) and interleukin-6 (IL-6) in LPS-injected mice (Figure 8A,B). The mRNA expression levels of both cytokines in kidney tissues were also significantly reduced by apamin (Figure 8C). Further, apamin reduced TLR4 expression and inhibited nuclear factor-κB (NF-κB) p65 phosphorylation (Figure 8D,E).

**Figure 9:**
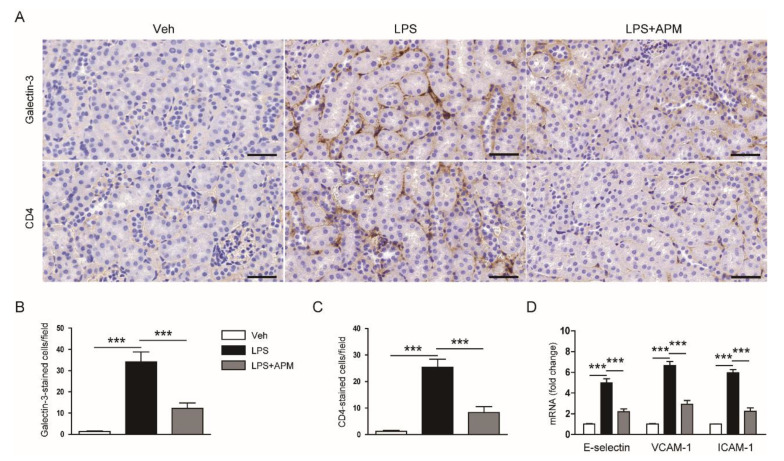
Effect of apamin on immune cell accumulation and expression of vascular adhesion molecules in LPS-injected mice. (**A**) Immunohiotochemical staining of kidney tissues for galectin-3 or CD4. Scale bars: 20 μm. (**B**) Number of galectin-3-stained cells. (**C**) Number of CD4-stained cells. (**D**) The mRNA expression of E-selectin, vascular cell adhesion molecule-1 (VCAM-1), and intercellular adhesion molecule-1 (ICAM-1). *n* = 8 per each group. *** *p* < 0.001.

Immune cell infiltration into the injured kidneys further aggravates LPS-induced inflammatory responses [2,3]. Thus, we next performed immunohistochemical staining of kidney sections with an antibody against galectin-3 or cluster of differentiation 4 (CD4) to identify macrophages and CD4^+^ T cells. We showed that apamin significantly inhibited the accumulation of macrophages and CD4^+^ T cells in LPS-injected mice, as reflected by reduced number of cells stained with antigalectin-3 or anti-CD4 antibody (Figure 9A–C). During sepsis, vascular adhesion molecules drive the infiltration of various immune cells into inflamed tissues [2,3]. We also found that increased mRNA levels of E-selectin, vascular cell adhesion molecule-1 (VCAM-1), and intercellular adhesion molecule-1 (ICAM-1) were significantly reduced by apamin (Figure 9D).

## 3. Discussion

Recently, we reported the beneficial effect of bee venom on LPS-induced renal injury [25]. However, whether apamin has a therapeutic effect on LPS-induced renal injury has not yet been clarified. In this study, we demonstrated that the administration of apamin after LPS injection effectively ameliorates LPS-induced renal dysfunction and histological injury, indicating that apamin has a therapeutic effect against LPS-induced AKI. These effects were attributed to inhibition of oxidative stress, apoptosis, and inflammation. Although previous studies have shown the beneficial effect of apamin against several inflammatory diseases [13,14,15,16,17,18], our study is the first to demonstrate the therapeutic action of apamin on LPS-induced AKI.

Although there remains some controversy about the role of tubular cell apoptosis in sepsis-associated organ damage, apoptosis has been recognized an important pathogenic mechanism in septic AKI [2,3]. During sepsis, PAMPs, such as LPS, activate immune cells and renal tubular epithelial cells via interaction with pattern recognition receptors, especially TLRs. These interactions result in an increase of cytokine synthesis and ROS generation in the cells. Increased oxidative stress can lead to mitochondrial injury and apoptotic death of tubular epithelial cells [2,3]. A previous study conducted on post-mortem kidney biopsy showed that prominent tubular apoptosis was detected in septic AKI patients, whereas apoptosis was barely observed in nonseptic AKI patients [26]. In addition, several substances have been reported to exert therapeutic effects in a cecal ligation and puncture (CLP) mouse model primarily through inhibiting tubular cell apoptosis [27,28]. Tubular cell apoptosis was also observed in LPS-injected rodents [29,30], where the administration of a pan-caspase inhibitor was shown to ameliorate LPS-induced AKI [31]. In our study, LPS treatment increased the amounts of lipid peroxidation products and reversed a reduction in GSH/GSSG ratio in the kidneys. These results are consistent with previous studies showing that markedly elevated levels of ROS, protein oxidation, and lipid peroxidation were detected in patients with septic AKI [20,32] and mice injected with LPS [19,20]. Interestingly, we found that oxidative stress induced by LPS was effectively alleviated by apamin. TUNEL staining and immunoblotting also showed that LPS treatment markedly induced tubular cell apoptosis in the kidneys, which was significantly alleviated by apamin. Consistent with our findings, a previous study showed that the administration of apamin attenuated apoptotic death of pancreatic acinar cells in an acute pancreatitis mouse model [17]. The antiapoptotic effect of apamin was also demonstrated in a rodent model of atherosclerosis induced by LPS and high-fat diet [33]. Collectively, these findings suggest that the therapeutic effect of apamin on LPS-induced renal injury is attributable to its antioxidative and antiapoptotic actions.

Current evidence suggests that NOX4 mainly contributes to ROS production and oxidative stress in various kidney diseases, including AKI [34]. The antioxidant enzyme HO-1 also plays an important role in LPS-induced renal injury [23,24]. Here, we observed that LPS-injected mice exhibited increased expression of NOX4 in the kidneys, which is consistent with previous studies [21,22]. In addition, HO-1 expression was also increased in LPS-injected mice compared to control mice. Similarly, recent studies also showed that LPS treatment led to HO-1 upregulation in the kidneys [35,36,37]. HO-1 catalyzes the degradation of heme to produce biliverdin, carbon monoxide, and ferrous iron. Biliverdin further degraded to bilirubin, which has antioxidative properties [23,24]. Thus, the upregulation of HO-1 induced by LPS may be a protective mechanism against oxidative stress. Interestingly, we found that the administration of apamin significantly attenuated NOX4 expression in LPS-injected mice. Apamin also further enhanced HO-1 expression. Therefore, these modulatory effects of apamin on NOX4 and HO-1 expression may primarily contribute to suppressing oxidative stress induced by LPS.

Inflammation also plays a critical role in septic AKI [2,3]. LPS is derived from gram-negative bacteria and activates multiple types of cells through binding to TLR4. Current evidence suggests that the TLR4 signaling pathway is critically involved in the inflammatory response in the CLP sepsis model [38,39] and the LPS-induced AKI model [40,41]. In addition, immune cell infiltration into injured kidneys has been known to aggravate inflammation and tissue injury [2,3]. Infiltration of multiple pro-inflammatory cells, such as macrophages and CD4^+^ T cells, is also commonly detected in the CLP sepsis model [39,42] and the LPS-induced AKI model [19,43]. Here, we showed that LPS-injected mice displayed increased expression of TLR4 compared to control mice. However, the administration of apamin significantly suppressed upregulation of TLR4 and reduced plasma and renal levels of cytokines in LPS-injected mice. Increased accumulation of macrophages and CD4^+^ T cells after LPS injection was also significantly attenuated by apamin. These results are consistent with previous studies showing the suppressive effect of apamin on cytokine production and immune cell accumulation in animal models of acute pancreatitis [17] and atherosclerosis [18]. Recently, we and others also reported that apamin inhibited cytokine production in LPS-treated microglial cells [44], cytokine-treated human keratinocytes [45], and airborne fungi-stimulated primary nasal fibroblasts [46]. It is known that vascular adhesion molecules are expressed in vascular endothelial cells, and that they facilitate the adhesion of immune cells to the endothelium and their extravasation into the tissues [47]. In this study, we also found that apamin significantly suppressed the elevated expression of E-selectin, VCAM-1, and ICAM-1. This result is in a good agreement with our previous study showing that apamin reduced expression of VCAM-1 and ICAM-1 in the vascular endothelium from atherosclerotic mice [18]. Altogether, these results suggest that apamin inhibits LPS-induced inflammatory responses via suppression of TLR4 signaling pathway and downregulation of vascular adhesion molecules.

## 4. Materials and Methods

### 4.1. Materials

Apamin (purity: ≥95%) and LPS were purchased from Sigma-Aldrich (St. Louis, MO, USA). A BUN assay kit was acquired from Thermo Fisher Scientific (Waltham, MA, USA) and a creatinine assay kit was obtained from Bioassay Systems (Hayward, CA, USA) respectively. The Glutathione Detection Kit was obtained from Enzo Life Sciences (Farmingdale, NY, USA). Enzyme-linked immunosorbent assay (ELISA) kits for TNF-α and IL-6 were purchased from R&D Systems (Minneapolis, MN, USA). The lipid peroxidation colorimetric/fluorometric assay kit was obtained from Sigma-Aldrich (St. Louis, MO, USA). The FITC-conjugated LTL was acquired from Vector Laboratories (Burlingame, CA, USA). Primary antibodies against cleaved caspase-3, cleaved PARP-1, NF-κB p65, p-NF-κB p65, or glyceraldehyde-3-phosphate dehydrogenase (GAPDH) were obtained from Cell Signaling (Danvers, MA, USA). Primary antibodies against KIM-1, 4-HNE, galectin-3, or CD4 were purchased from Abcam (Cambridge, MA, USA). Primary antibodies against NGAL or TLR were acquired from Santa Cruz Biotechnology (Santa Cruz, CA, USA). The primary antibody against NOX4 was obtained from Novus Biologicals (Littleton, CO, USA) and the primary antibody against HO-1 was purchased from Enzo Life Sciences (Farmingdale, NY, USA). The TRIzol reagent was acquired from Thermo Fisher Scientific (Waltham, MA, USA). The RNA to cDNA EcoDry™ Premix Kit, the RNA to cDNA EcoDry™ Premix Kit and the Power SYBR Green PCR Master Mix were obtained from TaKaRa (Tokyo, Japan). The in situ cell death detection kit was obtained from Roche Diagnostics (Indianapolis, IN, USA).

### 4.2. Animals

Animal experiments were carried out on male C57BL/6N mice (8 weeks of age; HyoSung Science Inc., Daegu, Korea). Mice were housed at 22 ± 2 °C in 12-h day/night cycles and had free access to standard rodent diet and water. The Institutional Animal Care and Use Committee of the Daegu Catholic University Medical Center approved all experimental procedures (Approval number: DCIAFCR-200507-03-Y).

### 4.3. Protocol of LPS-Induced AKI

Mice were arbitrarily categorized into three groups (*n* = 8 per each group): a vehicle-treated group (Veh), an LPS-injected group (LPS) and a group treated with LPS plus apamin (LPS + APM). Mice in the LPS group were intraperitoneally injected with LPS (10 mg/kg). Mice in the Veh group were subjected to an intraperitoneal injection of an equal volume of 0.9% saline. Apamin (0.1 mg/kg) was intraperitoneally administered to mice in the LPS + APM group 1 h after LPS injection. The doses of apamin and LPS used in the present study were chosen based on the results of previous studies [16,19]. At 24 h after LPS injection, all mice were sacrificed. Kidneys were promptly isolated and blood sample collection was carried out by cardiac puncture.

### 4.4. Biochemical Analyses

Separation of whole blood into plasma was carried out using a centrifugation method. Renal function was assessed by evaluating plasma concentrations of creatinine and BUN, which were performed using creatinine and BUN assay kits, respectively, according to the manufacturer′s protocol. Plasma concentrations of TNF-α and IL-6 were analyzed using ELISA kits according to the manufacturer′s protocol. Amounts of malondialdehyde (MDA), a lipid peroxidation product, in kidneys were analyzed using a lipid peroxidation colorimetric/fluorometric assay kit according to the manufacturer′s protocol. The GSH/GSSG ratio in kidneys was measured using the Glutathione Detection Kit according to the manufacturer’s protocol.

### 4.5. Histological Analysis, Immunohistochemical Staining, and Immunofluorescent Staining

Kidneys were fixed in 4% paraformaldehyde. Then, the tissues were dehydrated, cleared, embedded in paraffin blocks, and sectioned at 4 μm. After sectioning, H&E and PAS stains were performed on the thin sections. Images were visualized and captured using a confocal microscope (Nikon, Tokyo, Japan). Tubular injury was assessed in five arbitrarily chosen fields (400× magnification) of PAS-stained sections for each kidney. The degree of injury was scored based on the percentage of injured area: 0, 0%; 1, ≤10%; 2, 11–25%; 3, 26–45%; 4, 46–75%; and 5, 76–100% [48,49].

For immunohistochemial staining, the sections were probed with primary antibodies against NGAL, KIM-1, galectin-3, CD4, or 4-HNE overnight. After washing, the sections were incubated with a secondary antibody. Hematoxylin was used as the nuclear counterstain. Images were visualized and captured using a confocal microscope (Nikon, Tokyo, Japan). The percentage of positive staining for specific targets was analyzed in five arbitrarily selected fields (×400 magnification) for each kidney using the i-Solution DT software (IMTechnology, Vancouver, BC, Canada). The number of cells stained with antigalectin-3 or CD4 antibody was computed in five arbitrarily selected fields (×400 magnification) for each kidney.

The brush borders of proximal tubules were identified by staining with FITC-conjugated LTL. Nuclei were stained with 4’, 6-diamidino-2-phenylindole (DAPI). Images were visualized and captured using a confocal microscope (Nikon). The percentage of LTL-stained areas was analyzed in five arbitrarily selected fields (×400 magnification) for each kidney.

### 4.6. Immunoblot Analysis

Proteins were extracted from tissues with a lysis buffer and were subjected to sodium dodecyl sulfate-polyacrylamide gel electrophoresis. Resolved proteins were transferred onto a nitrocellulose membrane. The membrane was probed with primary antibodies against NGAL, NOX4, HO-1, cleaved caspase-3, cleaved PARP-1, TLR4, NF-κB p65, p-NF-κB p65, or GAPDH, followed by incubation with horseradish peroxidase-conjugated secondary antibodies. GAPDH were used as a loading control. The iBright™ CL1500 Imaging System (Thermo Fisher Scientific, Waltham, MA, USA) was used for detecting chemiluminescent signals.

### 4.7. Real-Time Reverse Transcription-Polymerase Chain Reaction (RT-PCR)

Total RNA was isolated from kidney samples using the TRIzol reagent (Thermo Fisher Scientific, Waltham, MA, USA). The RNA to cDNA EcoDry™ Premix Kit was used to synthesize cDNA. Real-time RT-PCR was conducted using the Power SYBR Green PCR Master Mix and the Thermal Cycler Dice Real Time System III (TaKaRa). Primers used in the present study are listed in Table 1. The internal reference gene was GAPDH.

### 4.8. TUNEL Assay

TUNEL staining was carried out to identify tubular cell apoptosis in the kidney sections using the in situ cell death detection kit according to the manufacturer’s protocol. Nuclei were stained with DAPI. Images were visualized and captured using a confocal microscope (Nikon). Cells stained with TUNEL were counted in five arbitrarily chosen fields (×400 magnification) for each kidney.

### 4.9. Statistical Analysis

Data are presented as mean ± standard error of the mean (SEM). One-way analysis of variance (ANOVA) with Bonferroni’s post hoc tests was used for comparison between groups. A *p* value less than 0.05 was considered statistically significant.

## 5. Conclusions

In conclusion, these results have demonstrated that apamin exerts antioxidative, antiapoptotic, and anti-inflammatory activities to ameliorate LPS-induced renal dysfunction and histological injury. Our findings suggest that apamin might be a useful therapeutic agent for septic AKI.

## Figures and Tables

**Table 1:** List of primers used in this study.

Gene	Primer Sequence (5′→3′)	Accession No.
NOX4 ^1^	Forward: GAACCCAAGTTCCAAGCTCATT Reverse: GGCACAAAGGTCCAGAAATCC	NM_015760
HO-1 ^2^	Forward: TGCAGGTGATGCTGACAGAGG Reverse: GGGATGAGCTAGTGCTGATCTGG	NM_010442
TNF-α ^3^	Forward: GACGTGGAACTGGCAGAAGAG Reverse: CCGCCTGGAGTTCTGGAA	NM_013693
IL-6 ^4^	Forward: CCAGAGATACAAAGAAATGATGG Reverse: ACTCCAGAAGACCAGAGGAAAT	NM_031168
E-selectin	Forward: AGCTACCCATGGAACACGAC Reverse: ACGCAAGTTCTCCAGCTGTT	NM_011345
VCAM-1 ^5^	Forward: CCCAGGTGGAGGTCTACTCA Reverse: CAGGATTTTGGGAGCTGGTA	NM_011693
ICAM-1 ^6^	Forward: TTCACACTGAATGCCAGCTC Reverse: GTCTGCTGAGACCCCTCTTG	NM_010493
GAPDH ^7^	Forward: ACTCCACTCACGGCAAATTC Reverse: TCTCCATGGTGGTGAAGACA	NM_001289726

^1^ Nicotinamide adenine dinucleotide phosphate oxidase 4. ^2^ Heme oxygenase-1. ^3^ Tumor necrosis factor-α. ^4^ Interleukin-6. ^5^ Vascular cell adhesion molecule-1. ^6^ Intercellular adhesion molecule-1. ^7^ Glyceraldehyde-3-phosphate dehydrogenase.
